# Clinical signs and anatomical correlation of patellar tendinitis

**DOI:** 10.4103/0019-5413.69317

**Published:** 2010

**Authors:** Ehud Rath, Ran Schwarzkopf, John C Richmond

**Affiliations:** Department of Orthopaedics, Soroka Medical Center, Ben Gurion University, Beer Sheva, Israel; 1 Department of Orthopaedics, NYU Hospital for Joint Diseases, New York, NY, USA; 2 Department of Orthopaedics, New England Baptist Hospital, Boston, Massachusetts, USA

**Keywords:** Anterior knee pain, patellar tendinitis, patellar tendon

## Abstract

**Background:**

Patellar tendinitis is one of the several differential diagnosis of anterior knee pain. The clinical diagnosis of patellar tendinitis is based on tenderness to palpation at the inferior pole of the patella. The tenderness has been noted to be maximal when the knee is extended and the quadriceps relaxed, but a definite clinical sign for diagnosis is lacking. The accuracy of two clinical signs was assesed by a two-stage study which included physical examination, MRI and a cadaveric study.

**Materials and Methods:**

Two clinical signs, the “passive flexion-extension sign” and the “standing active quadriceps sign” were assessed in 10 consecutive patients with presumed patellar tendinitis. Five patients had an MRI, showed focal abnormality in the tendon. The location of the MRI finding corresponded, to the region of maximal tenderness. A cadaveric dissection was undertaken to describe the anatomy of the patella and the patellar tendon during these tests.

**Results:**

Both tests showed a significant decrease in tenderness at the area of inflammation when the patellar tendon was under tension. The cadaveric dissection showed that when the knee is flexed to 90° or when the quadriceps is tensioned the deep fibers of the tendon do not deform to anteriorly applied pressure.

**Conclusion:**

We suggest using these studies routinely in the evaluation of patients with anterior knee pain.

## INTRODUCTION

Patellar tendinitis or jumper’s knee is a common overuse injury in sports. First described by Blazina 1 in 1973, the condition develops in athletes who engage in various sports, particularly those that lead to high eccentric quadriceps loading such as basketball, volleyball, long distance running, and skiing.^2^–^4^ The proximal part of the patellar tendon is most often affected and the proximal third of the tendon is usually thickened.^5^

Patellar tendinitis is one of the several differential diagnosis of anterior knee pain. The diagnosis can be mistaken for other disorders or injuries, such as bursitis, meniscal tear, chondromalacia or other causes of the patellofemoral pain syndrome.^2^ A detailed history and a careful physical examination often leads to the correct diagnosis, but surprisingly, the literature lacks for specific clinical signs to diagnose the problem.

The purpose of this paper is to assess the two clinical signs used routinely at our clinic for patellar tendinitis. The reproducibility of these signs was evaluated on 10 consecutive patients and a second stage cadaveric study was performed to show an anatomic correlation for these signs.

## MATERIALS AND METHODS

Ten consecutive patients with anterior knee pain confined to the inferior pole of the patella and the proximal patellar tendon were included. There were six females and four males. Average age was 30.3 (range 22 years-47 years) years. In seven patients the symptoms were related to athletic activity (six - long distance running, one - soccer). None had previous injury, injection, or surgery to the affected knee.

### Clinical signs

**Figure 1:**
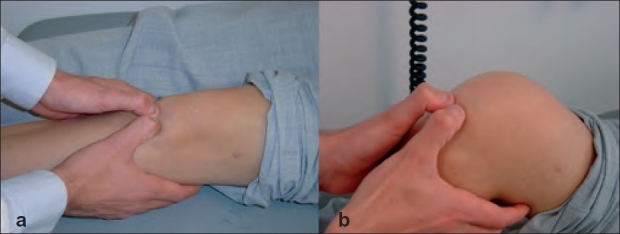
Clinical photograph showing (a) Palpation of the patellar tendon in passive extension (b) Palpation of the patellar tendon in 90° flexion

Two clinical signs were performed to assess patellar tendinitis. In the “passive extension – flexion sign” the patient lies supine on the examination table. The anterior aspect of the extended knee is palpated to define the point of maximal tenderness [Figure 1a]. In the case of patellar tendinitis, tenderness to palpation of the tendon is most often located at the origin of the tendon at the inferior pole of the patella. Once the point of maximal tenderness is identified, the knee is flexed to 90° and pressure is again applied to the tendon [Figure 1b].

**Figure 2:**
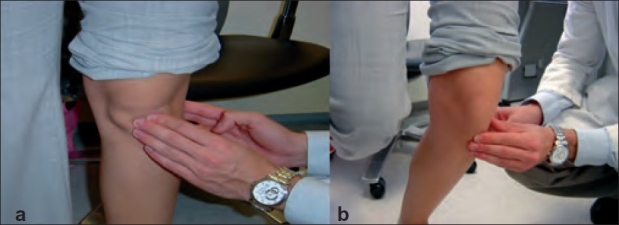
Clinical photograph showing (a) Standing active quadriceps test, weight bearing in full extension (b) Standing active quadriceps test, weight bearing in 30° of flexion

For the “standing active quadriceps sign”, the patellar tendon is palpated along its course while the patient stands [Figure 2a]. The point of maximal tenderness identified. The patient is then asked to stand only on the involved extremity with 30° of knee flexion and the tendon was re-palpated [Figure 2b].

In both these tests, the patient should note a marked reduction of tenderness to palpation when the knee is flexed or the quadriceps contract, in order to confirm the diagnosis of patellar tendinitis.

### Patient evaluation

A visual analog pain scale was used to describe the level of pain provoked as the tendon was palpated. 0 on the scale is no pain experienced at all and 10 is the worst pain the patient ever experienced.

Magnetic resonance imaging (MRI) was performed on five patients to evaluate presence of signs for tendinitis. For statistical analysis, the paired T-test was used to compare the visual analog pain scale of each patient on the two parts of each test. Significance was set at *P*<0.05.

### Cadaveric dissection

Exploration of the extensor mechanism was carried out on a single specimen. Through a midline incision, the quadriceps tendon, patella, and whole patellar tendon were exposed. The patella was cut sagitally using an oscillating saw. The patellar tendon was then sharply split in half down to the tibial tuberosity. The dissected half was amputated to facilitate visualization of the tendon fibers. Careful observation was applied to the orientation of the proximal fibers of the patellar tendon on the various positions of the described tests.

## RESULTS

The location of tenderness was at the tendon origin from the inferior pole of the patella in all 10 patients. All the patients had marked reduction of tenderness to palpation in the flexed knee position or with the quadriceps contracted. The visual analog pain scale showed statistical significant reduction of pain with flexion or quadriceps contraction (*P*<0.0001). The average score of the untensed tendon was 5 compared to 2 on the tensed patellar tendon (*P*<0.001).

**Figure 3:**
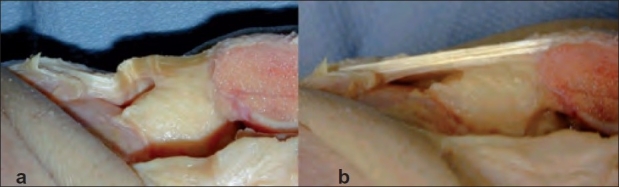
Cadaveric dissected specimen showing (a) The patellar tendon without tension (b) The patellar tendon under tension (Notice how the tensed anterior fibers could serve as a barrier to resist applied pressure anteriorly and to protect the posterior part of the tendon)

Of the five patients who underwent MRI of the involved knee, all showed high signal intensity within the proximal posterior central aspect of the tendon at its origin. The cadaveric dissection showed the whole tendon to be loose in extension [Figure 3a]. The posterior aspect of the tendon was easily deformed under anteriorly applied pressure. As the knee was flexed to 90° or the quadriceps tensioned, the stretched anterior fibers protected the posterior fibers from deformation by pressure under the same force anteriorly applied and posteriorly directed [Figure 3b].

## DISCUSSION

Though many papers have been written on patellar tendinitis, none of them has focused on the clinical diagnosis or studied a specific sign. Khan *et al*.^3^ in a recent paper were the first to describe the key clinical sign in patellar tendinitis, the passive extension flexion test. We are aware of no prior anatomic study to demonstrate the basis of these clinical tests.

The suggested pathogenesis of patellar tendinitis is impingement between the deep fibers of the proximal patellar tendon on the inferior pole of the patella in flexion.^5^ This theory explains the characteristic site of the lesion – at the very proximal, middle, and posterior aspect of the tendon.

Ultrasound and MRI are considered the imaging modalities of choice in patients with tendon disorders. However, Johnson *et al*^5^ show that the inflammatory lesion seen on MRI scans is less reliable indicator of patellar tendinitis than increased tendon thickness. Further, Shalaby and Almekinders^6^ showed that in younger patients with relatively mild symptoms, MRI did not show significant changes, while in older, active patients changes may be present in asymptomatic knees. These findings stress the importance of reproducible and simple clinical signs for patellar tendinitis. The signs discussed in this paper meet these criteria and we suggest using them routinely in the clinical assessment of anterior knee pain.
